# Dietary Restriction and Lipid Metabolism: Unveiling Pathways to Extended Healthspan

**DOI:** 10.3390/nu16244424

**Published:** 2024-12-23

**Authors:** Hye-Yeon Lee, Kyung-Jin Min

**Affiliations:** Department of Biological Sciences and Bioengineering, Inha University, Incheon 22212, Republic of Korea; lee.hy@inha.ac.kr

**Keywords:** dietary restriction, lipid profile, healthspan, sirtuin, target of rapamycin, Ins/IGF-1, cell membrane, commensal bacteria

## Abstract

Dietary restriction (DR) has been reported to be a significant intervention that influences lipid metabolism and potentially modulates the aging process in a wide range of organisms. Lipid metabolism plays a pivotal role in the regulation of aging and longevity. In this review, we summarize studies on the significant role of lipid metabolism in aging in relation to DR. As a potent intervention to slow down aging, DR has demonstrated promising effects on lipid metabolism, influencing the aging processes across various species. The current review focuses on the relationships among DR-related molecular signaling proteins such as the sirtuins, signaling pathways such as the target of rapamycin and the insulin/insulin-like growth factor (IGF)-1, lipid metabolism, and aging. Furthermore, the review presents research results on diet-associated changes in cell membrane lipids and alterations in lipid metabolism caused by commensal bacteria, highlighting the importance of lipid metabolism in aging. Overall, the review explores the interplay between diet, lipid metabolism, and aging, while presenting untapped areas for further understanding of the aging process.

## 1. Introduction

Dietary restriction (DR), a dietary regimen characterized by reduced calorie intake without causing malnutrition, has emerged as a significant intervention that influences lipid metabolism and potentially modulates the aging process in a wide range of organisms including yeast, nematodes, fruit flies, rodents, and primates [1]. Many studies have demonstrated that DR delays aging via the sirtuin (SIRT), target of rapamycin (TOR), and insulin/insulin-like growth factor-1 (ins/IGF-1) signaling pathways [2,3]. These pathways are well known to be involved in lipid metabolism, thereby suggesting the role of lipid metabolism in aging [4,5]. Evidence corroborating this notion is discernible when examining aging-related research on compounds identified as potential calorie restriction mimetics, such as resveratrol, rapamycin, and metformin, which reveal an association between energy metabolism and the aging process [4,5,6]. Resveratrol, a typical DR mimetic, is known to not only regulate the biosynthesis of the adipocyte life cycle and fat [7] but also to enhance fat degradation in adipocytes cultured in vitro [8]. In addition, studies on mistletoe, which has been reported to increase the lifespan in yeast, nematodes, fruit flies, and silkworms, have shown that mistletoe increased sensitivity to starvation in fruit flies [9], reduced body weight in the silkworm moth [10], and inhibited fatty acids biosynthesis [11]. This suggests that the lifespan-extending mechanism of DR mimetics is correlated with energy storage.

It is currently suggested that the human lifespan is about 120 years [12]. Jeanne Calment (1875–1997), officially the world’s longest-living person, was recorded as living for 122 years and 164 days (The Guinness Book of World Records 1999). The World Population Ageing 2009 report by the United Nations Department of Economic and Social Affairs (UN DESA) used the term ‘Homo hundreds’ to indicate that Homo sapiens (humans) can live for over 100 years. In addition, improvements in medicine, economics, and technology have improved the quality of life of human beings, and accordingly, ’food intake’ has been associated with improved life quality in people today. Predominantly carbohydrate-based diets have now been replaced by high-fat-based diets in recent times. Also, the excessive intake of proteins and carbohydrates leads to excess energy, which is ultimately converted to the lipid form and stored in the body. This excessive intake of energy, including fat, has been reported to increase the incidence of metabolic diseases such as obesity, diabetes, cardiovascular disease, and cancer [13,14,15,16]. Thus, diet-based lipid metabolism in the body is closely related to the aging of organisms. This report reviews perspectives on the relationship between DR, lipid metabolism, and aging.

## 2. Molecular Mechanisms of Dietary Restriction Regulating Lipid Metabolism

**Figure 1:**
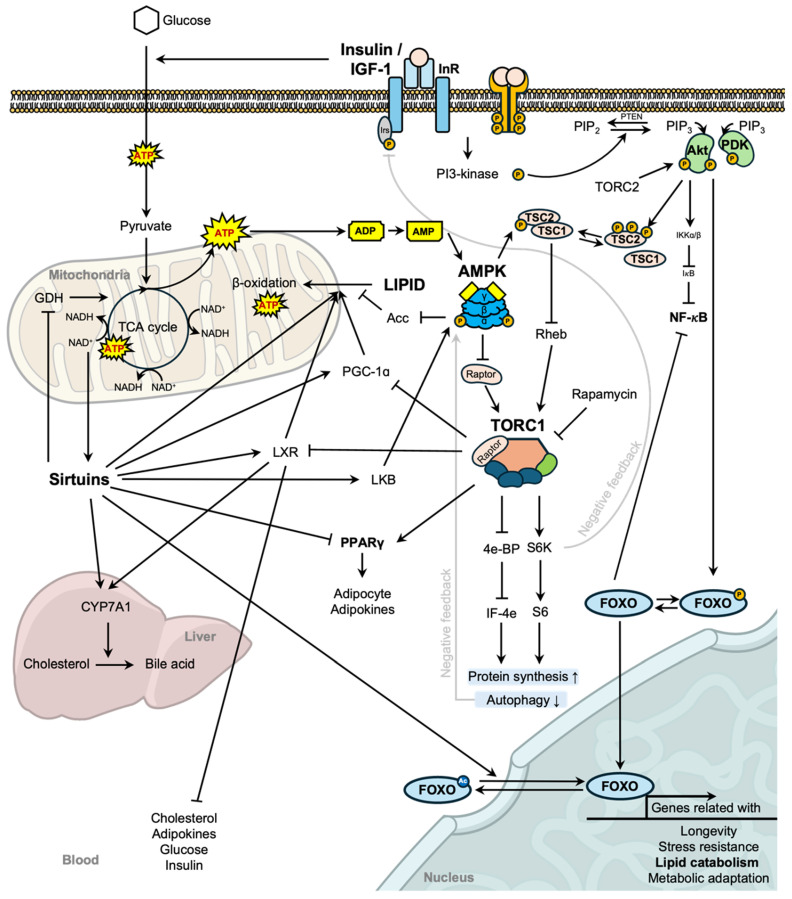
Molecular mechanisms linking dietary restriction to lipid metabolism and health benefits. The figure illustrates the molecular pathways influenced by dietary restriction (DR) and their effects on lipid metabolism and overall health. Key pathways include NAD^+^-dependent sirtuin signaling, the TOR pathway, AMPK activation, and insulin/IGF-1 signaling, which collectively regulate metabolic processes. By reducing energy intake, DR enhances mitochondrial β-oxidation, promoting the breakdown of fatty acids to generate energy, while increasing NAD^+^ levels to activate SIRT1, further driving lipid catabolism. DR also suppresses insulin/IGF-1 signaling by activating the TSC1/2 complex to inhibit mTORC1, shifting the cellular focus from anabolic processes to lipid utilization and autophagy. Additionally, FOXO transcription factors, activated by DR, upregulate genes involved in lipid breakdown and metabolic adaptation, supporting efficient fat utilization and stress resistance. In the liver, DR enhances cholesterol metabolism by activating CYP7A1, facilitating the conversion of cholesterol into bile acids to maintain lipid homeostasis. Furthermore, DR reduces circulating levels of cholesterol, adipokines, glucose, and insulin, promoting lipid catabolism, metabolic adaptation, stress resistance, and longevity. These coordinated changes highlight DR’s critical role in optimizing energy balance, lowering the risk of metabolic diseases, and extending the healthspan.

DR is a proven intervention that extends the lifespan and enhances metabolic health across species, from yeast to mammals [3]. The interest in DR research is its influence on conserved cellular pathways such as the sirtuin, TOR, and insulin/IGF-1 signaling pathways, which regulate metabolism, energy homeostasis, stress responses, and longevity (Figure 1). Under DR conditions, elevated nicotinamide adenine dinucleotide (NAD^+^) levels enhance sirtuin activity, particularly SIRT1. NAD^+^-dependent deacetylation is a process crucial for sensing the energy status of the cell. NAD^+^ levels are indicative of cellular energy balance, and the consumption of NAD^+^ influences directly the cellular survival. This activation promotes metabolic adaptation through the deacetylation of key proteins, including AMP-activated protein kinase (AMPK), leading to increased energy efficiency and lipid oxidation. Sirtuins also modulate the activity of transcription factors such as Forkhead box-O and p53, enhancing stress resistance, DNA repair, and apoptosis regulation. TOR pathway, a critical nutrient-sensing mechanism that regulates growth, metabolism, and lifespan, is inhibited under the conditions of DR. Reduced nutrient availability leads to decreased activation of Akt, which in turn diminishes mammalian TOR complex 1 (mTORC1) activity. mTORC1, which includes TOR and raptor, is negatively regulated by the TSC1/TSC2 complex. Since TORC1 activation phosphorylates downstream effectors such as S6 kinase (S6K) and 4E-binding protein (4E-BP), lowered TORC1 activity downregulates S6K activation and upregulates 4E-BP, resulting in reduced protein synthesis and enhanced cellular stress resistance. These changes contribute to an increase in autophagy and a shift from anabolic processes to catabolic pathways, ultimately supporting cellular maintenance and promoting lifespan extension. The insulin/IGF-1 pathway is reduced under DR, leading to decreased signaling through phosphoinositide 3-kinase (PI3K) and phosphoinositide-dependent kinase (PDK). This cascade results in lower activation of Akt, which in turn prevents the phosphorylation and inactivation of FOXO transcription factors. Activated FOXO trans-localization to the nucleus, where it stimulates the expression of genes involved in stress resistance, lipid catabolism, and metabolic adaptation. These pathways play crucial roles in lipid metabolism, promoting fat oxidation, reducing fat storage, and modulating lipid synthesis. DR reduces the accumulation of fat in the liver, particularly under high-fat diet conditions, and improves mitochondrial efficiency in lipid metabolism. This suggests a protective role of DR in managing lipid disorders like non-alcoholic fatty liver disease [17]. In long-term DR diets during a 2-year period in Biosphere 2—a closed ecological system designed to study interactions between humans, agriculture, and the environment—significant reductions in total plasma cholesterol and triglyceride levels have been observed, demonstrating that DR prevents hyperlipidemia and improve lipid profiles, which are key factors in preventing cardiovascular diseases [18]. Interestingly, restricting specific dietary components, even without limiting overall diet, can also impact lipid profile. For instance, carbohydrate restriction can improve lipid metabolism by reducing triglycerides, low-density lipoprotein, and total cholesterol levels. It promotes lipid oxidation, improves insulin sensitivity, and reduces fat storage, contributing to improved cardiovascular health [19]. Also, restriction of dietary methionine increases fat oxidation and reduces liver lipid accumulation without affecting body weight, indicating a potential therapeutic role in lipid disorders independent of caloric intake [20]. In summary, DR beneficially impacts lipid profiles, reduces fat accumulation, enhances insulin sensitivity, and promotes fat oxidation, supporting its potential as a strategy to prevent and manage lipid disorders and metabolic diseases. However, long-term studies and further research are needed to fully understand its sustained impacts on lipid metabolism.

### 2.1. Sirtuins and Lipid Profile

Silent information regulator 2 (Sir2) proteins, or sirtuins, are highly conserved NAD^+^-dependent protein deacetylases present in a diverse range of organisms from yeasts to humans. Sir2 was first found to regulate replication-associated aging in the budding yeast, *Saccharomyces cerevisiae* [21]. In response to cellular energy states, it plays a role in activating the metabolism via metabolic enzymes, as well as histones and transcription factors [22]. Activation of sirtuins reduces blood cholesterol, adipokines, glucose, and insulin [23], and prolongs lifespan by managing energy homeostasis and stress [24,25]. The sirtuin-activating mechanism is involved in the lifespan-increasing effect of DR. In addition, sirtuin directly or indirectly affects lipid metabolism in white and brown adipose tissue (WAT and BAT), the liver, and skeletal muscle by modulating key metabolic pathways. The sirtuin family in mammals, encompassing Sirtuin 1 to Sirtuin 7, plays pivotal roles in numerous cellular functions, including cell survival, aging, genomic integrity, and metabolic regulation [26]. Each sirtuin is activated in distinct locations and performs unique functions, with varying responses to CR. For example, CR or fasting upregulates Sirtuin 1 [27], Sirtuin 2 [28], Sirtuin 3 [29], and Sirtuin 6 [30] while downregulating Sirtuin 4 [31] in adipose tissue, with no significant effect on Sirtuin 7 [32] expression. The sirtuin family not only acts as crucial sensors of cellular energy status but also confers protection against metabolic challenges. Sirtuin 1 (SIRT1) reduces adipocyte production in white adipocytes by inhibiting the transcriptional activity of nuclear receptor peroxisome proliferator-activated receptor γ (PPARγ), a major ligand-active transcription factor that regulates adipocyte differentiation [33]. Interestingly, SIRT1 causes the promoters of the PPARγ target gene to aggregate in starvation conditions [34] and regulates adipocyte production and function in response to starvation or DR [35]. In addition, PPARγ, PPARγ coactivator 1α (PGC-1α), and the liver X receptor (LXR) in the liver, which is the main organ responsible for fatty acid, cholesterol, and bile acid synthesis and lipoprotein absorption, synthesis, and secretion, increase the targeting of LXR and β-oxidation [36,37,38]. The skeletal muscle also promotes β-oxidation through the deacetylation of PGC-1α [39,40]. The promotion of the β-oxidation of free fatty acids plays an important role in producing adenosine triphosphate (ATP) and providing it to the brain under starvation conditions. ATP consumption increases the intracellular adenosine monophosphate (AMP)/ATP ratio and activates AMP-activated protein kinase (AMPK), which stimulates fatty acid oxidation, triglyceride accumulation, and mitochondrial biosynthesis [41]. SRIT1 also stimulates the activity of AMPK by deacetylating liver kinase B1 (LKB1), which is an activation factor of AMPK. In this way, SIRT1 and AMPK regulate the mitochondrial oxidation of fatty acids in a nutrient-limited manner. This effect is observed both in vitro and in vivo: for example, α-lipoic acid enhances SIRT1-mediated fat oxidation and reduces fat storage in cells and diabetic mice [42]. Sirtuin 2 (SIRT2) indirectly affects white adipose tissue by the deacetylation of the transcription factor Forkhead box O1 (FoxO1) protein. Even though the process of SIRT2 differs from that of SIRT1, it results in reducing the production of adipocytes in white adipose tissue and promoting lipolysis. Unlike SIRT2, sirtuin 3 (SIRT3) is involved in brown adipose tissue. The expression of SIRT3 in brown adipocytes not only increases the expression of PGC-1α, the main regulator of mitochondrial biosynthesis gene expression, but also increases the respiration rate through the activation of uncoupling protein 1 (UCP1) present in the mitochondrial inner membrane [29]. Also, SIRT3 enhances mitochondrial fatty acid oxidation, particularly in response to high fatty acid conditions, by regulating enzymes involved in the breakdown of fatty acids. This reduces lipid accumulation in liver cells and improves energy homeostasis [43]. Enhanced mitochondrial function by SIRT3, activated through DR, plays a crucial role in promoting fatty acid oxidation, thereby regulating brain lipid metabolism and supporting overall mitochondrial health [44]. Some studies predict that the stimulation of brown fat thermogenesis by the pharmacological activation of sirtuins could be used as a new strategy for the treatment of obesity and related diseases [45,46]. Sirtuin 4 (SIRT4) regulates lipid homeostasis and metabolism by controlling insulin secretion [23]. Insulin is generally known only as a hormone that synthesizes glucose into glycogen. However, research on the relationship between insulin and aging is actively underway, as it became known that FOXO is regulated by insulin and insulin-like growth hormones. SIRT4 is expressed in the islets of Langerhans that inhibit glutamate dehydrogenase (GDH) through NAD^+^ dependent ADP-ribosylation [31]. GDH is a mitochondrial enzyme that promotes ATP production by inducing amino acids into the citric acid cycle by converting glutamate to α-ketoglutarate [31]. Islets of Langerhans isolated from SIRT4-deficient mice showed increased GDH activity and insulin secretion regardless of the glucose and amino acid levels [31]. The over-expression of SIRT4 in insulinoma cells has been shown to inhibit insulin secretion [47]. In addition, SIRT4 inhibits fatty acid oxidation by repressing malonyl CoA decarboxylase (MCD). This action increases malonyl CoA levels, which both promotes lipid synthesis and inhibits fat oxidation. Mice lacking SIRT4 showed elevated fat oxidation, highlighting its regulatory role in lipid balance [48]. Therefore, the modulation of SIRT4 activity can potentially provide a new therapeutic approach for the treatment of metabolic diseases. Sirtuin 5 (SIRT5), a mitochondria-localized sirtuin, plays a crucial role in regulating lipid metabolism and promoting fatty acid oxidation [49]. Studies using bovine preadipocytes and obese mice have shown that SIRT5 activates the AMPK pathway while repressing the MAPK pathway during adipocyte differentiation [50]. In Sirt5-knockout brown adipose tissue (BAT), reduced function of UCP1 and associated proteins leads to impaired mitochondrial respiration, defective mitophagy, and metabolic inflexibility [51]. Therefore, Sirt5 plays a critical role in maintaining energy homeostasis by regulating UCP1, a key thermogenic protein in BAT. Sirtuin 6 (SIRT6), in cooperation with SIRT5, reduces lipid deposition in bovine preadipocytes. It inhibits preadipocyte differentiation and lipid accumulation by activating the AMPKα pathway and suppresses adipose tissue differentiation and lipid synthesis in obese mice in vivo [52]. Fat-specific Sirt6-knockout mice are more susceptible to high-fat-diet-induced obesity and exhibit increased inflammation in adipose tissue. This effect is linked to the suppression of adipose triglyceride lipase (ATGL), a key lipolytic enzyme, caused by increased phosphorylation and acetylation of FoxO1, which impairs its transcriptional activation of ATGL. Similarly, in obese patients, reduced SIRT6 expression is associated with decreased ATGL expression [53]. A study investigating the role of SIRT6 in adipocytes found that fat-specific *Sirt6*-knockout mice showed impaired responses to intermittent fasting (IF), including glucose and insulin intolerance, reduced energy expenditure, impaired adipose tissue browning, and increased WAT inflammation. These effects were linked to reduced UCP1 expression and downregulation of p38 MAPK/ATF2 signaling. Interestingly, SIRT6 deficiency in hepatocytes or in myeloid cells did not impair adaptation to IF. The findings highlight that SIRT6 in adipocytes is essential for the metabolic benefits of IF [54]. Additionally, sirtuin 7 (SIRT7) regulates lipid metabolism by influencing the degradation of key proteins involved in lipid storage. *Sirt7*-knockout mice showed resistance to fatty liver and obesity induced by a high-fat diet, suggesting that SIRT7 promotes lipid accumulation through regulation of fatty acid uptake and triglyceride synthesis [55]. Interestingly, *Sirt7*- knockout mice exhibit a significant reduction in white WAT due to elevated SIRT1 activity, and inhibition of SIRT1 restored WAT levels in these mice [56]. In summary, notably, SIRT1, SIRT3, and SIRT6 are central regulators of adipogenesis, lipid mobilization, and anti-inflammatory responses and play prominent roles in adipose tissue browning and thermogenesis by repressing PPARγ and PGC-1α and promoting AMPK activation. SIRT2 and SIRT5 modulate adipocyte function through pathways involving FOXO1, PGC-1α, and lipolysis regulation. SIRT4 increases adipocyte differentiation by repressing fatty acid oxidation. SIRT7 promotes adipogenesis by suppressing SIRT1 activity while lacking significant involvement in lipid mobilization, inflammation, fibrosis, or browning. The sirtuin family is intricately involved in the coordination of autophagic processes. The role of autophagy in lipid metabolism represents a novel and promising field of study, potentially offering new perspectives on the metabolic regulation of lipids [57,58]. Various natural compounds with a sirtuin modulation function, such as curcumin, resveratrol, and berberine, have been identified as influencing metabolism targeting sirtuins to regulate autophagy [26,59,60]. Overall, there is still a need to elucidate the diverse mechanisms by which sirtuins regulate aging through lipid metabolism. Particularly considering recent research trends, studies related to autophagy are anticipated to be vigorously pursued in the future. Thus, a study on the role of sirtuins in lipid metabolism can offer promising targets for treating metabolic diseases and extending the lifespan.

### 2.2. Target of Rapamycin Pathway and Lipid Profile

Target of rapamycin (TOR) is a major nutrient-sensing mechanism that regulates metabolism and is well conserved from yeasts to humans [61]. Mammalian TOR (mTOR) may be described as a serine/threonine kinase that forms two complexes, mTORC1 and complex 2 (mTORC2), which regulate growth, survival, autophagy, and metabolism [62]. Importantly, mTORC1 activity is subject to intricate feedback regulation to maintain cellular homeostasis. For example, the activation of mTORC1 inhibits insulin receptor substrate (IRS) signaling via the S6K1-mediated phosphorylation of IRS proteins, which creates a negative feedback loop that inhibits PI3K-Akt signaling [63]. Similarly, mTORC1 activation can suppress autophagy by phosphorylating Unc-51-like autophagy activating kinase 1 (ULK1), yet prolonged nutrient deprivation leads to upstream AMPK activation, which inhibits mTORC1 and reactivates autophagy to restore energy balance [64]. These feedback mechanisms highlight the dynamic and tightly regulated nature of the TOR pathway in cellular metabolism. When there is an anabolic stimulus, mTORC1 and mTORC2 are instrumental in increasing triglyceride accumulation through the enhancement of the adipogenic and lipogenic pathways [62]. Activation of mTORC1 promotes de novo lipogenesis by upregulating key enzymes like sterol regulatory element-binding protein 1c (SREBP1c) and fatty acid synthase (FAS). Moreover, TOR activation inhibits the expression of ATGL and hormone-sensitive lipase (HSL), thus reducing fat breakdown. This leads to the accumulation of triglycerides and fat storage in tissues like the liver and adipose tissue [65,66]. Concurrently, these complexes orchestrate the efficient sequestration of energy by attenuating catabolic pathways, notably lipolysis and β-oxidation, thereby ensuring the conservation of energy reserves within the organism. For instance, the inhibition of mTORC1 by rapamycin, an inhibitor of TORC1, increases β-oxidation and the expression of enzymes involved in fatty acid oxidation, such as acyl-CoA dehydrogenase, very long chain (ACADVL) and carnitine-palmitoyltransferase-1 [67,68]. Rapamycin reduces the expression of lipogenic genes like glucokinase and FAS in rainbow trout, resulting in decreased lipid accumulation [69]. In yeast, rapamycin treatment leads to the replenishment of lipid droplets, highlighting its role in lipid homeostasis [70]. Moreover, mTORC1 can control ketogenesis in mice in response to fasting [71]. Sengupta et al. demonstrated that the liver-specific loss of tuberous sclerosis complex 1 (TSC1), a known inhibitory regulator of mTORC1, markedly impairs the capacity of liver cells to initiate β-oxidation in response to fasting conditions and suggested a role for mTORC1 activity in promoting the aging of the liver by regulating the PPARα function and hepatic ketogenesis. Specifically, it is well-known that the functions of mTORC1 respond to various environmental inputs, like growth factors, amino acids, and stress, to regulate cell growth and proliferation [50]. In an experiment examining the relationship between a high-fat diet and TOR, the inhibition of the TOR signaling pathway prevented an increase in triglycerides and reduced insulin signaling in fruit flies fed on a high-fat diet [72]. In addition, the TOR signal mediates pancreatic β-cell growth and functional nutrient responses (such as insulin and glucose) [73,74,75]. The elimination of the TOR effector, S6 kinase 1 (S6K1), can prevent the accumulation of fat after consuming high-fat diets [76,77]. Although the inhibition of mTORC1 via rapamycin treatment can affect lipolysis [65], there is no evidence of an indirect effect by mTORC2 regulation. The mTORC1 function could also be inhibited by rapamycin in rats and some cell types [78,79]. However, most of the studies investigating the effect of rapamycin have focused only on the mTORC1 function, but not on the role of mTORC2 [65,80,81,82,83]. Despite considerable progress in unraveling the mechanisms linking the mTOR complexes to lipid metabolism, further research is essential to comprehensively understand the specific actions and contributions of each mTOR complex in the regulation of lipid metabolic processes.

### 2.3. Insulin/Insulin-like Growth Factor 1 and Lipid Profile

The insulin/insulin-like growth factor 1 (IGF-1) signaling pathway, which is evolutionarily conserved across various species ranging from yeast to humans, plays a pivotal role in a host of interconnected functions essential for metabolism, growth, and reproduction [84]. The insulin/IGF-1 signaling (IIS) pathway plays a pivotal role in the regulation of longevity and is represented as a pathway downregulated by DR [3]. Insulin transports blood glucose into the cells, regulates cellular growth, and controls metabolism-related gene transcription. Insulin signaling is closely related to carbohydrate metabolism but is also known to play an important role in lipid metabolism, affecting various aspects such as lipid synthesis, storage, and breakdown [85,86,87]. For instance, insulin activates lipogenic pathways, particularly in the liver and adipose tissue. It enhances the expression and activity of key enzymes involved in lipid synthesis, such as acetyl-CoA carboxylase (ACC) and FAS, which leads to increased fat production and storage [88]. Simultaneously, insulin suppresses the breakdown of stored triglycerides in adipocytes by inhibiting the action of hormone-sensitive lipase and adipose triglyceride lipase [89,90]. This prevents the release of free fatty acids (FFAs) into the bloodstream, promoting fat retention in adipose tissue. Elevated FFAs can impair insulin signaling, leading to metabolic insulin resistance by inhibiting insulin-stimulated glucose uptake and suppressing nitric oxide production, which can affect vascular function [91,92]. This process is crucial for converting excess glucose into stored energy, reducing fatty acid levels in the blood, and improving energy utilization efficiency. Furthermore, insulin plays a pivotal role in energy production by regulating the oxidation of fatty acids in muscles and other tissues, ensuring a balanced interplay between energy expenditure and storage. Also, a high proportion of fatty acids such as linoleic acid in the diet is associated with impaired insulin sensitivity independent of obesity [93]. In insulin-resistant conditions (e.g., type 2 diabetes), patients have altered fatty acid composition, indicating that the insulin pattern can alter the lipid composition in the body [93], and insulin fails to suppress hepatic glucose production but continues to promote lipogenesis, leading to excessive fat accumulation in the liver, a phenomenon known as selective insulin resistance [94]. Furthermore, skeletal muscle is a critical factor in determining insulin sensitivity. Research has consistently linked insulin resistance with higher saturation levels of phospholipids in the skeletal muscle cell membrane [95]. Additionally, insulin signaling regulates the expression of acyl Co-A synthetases (ACS), which are responsible for activating triglyceride catabolism in mammalian cells [96]. In *Drosophila*, increased ACS expression, transcriptionally regulated by FOXO activation, causes a decrease in triglyceride levels, indicating that ACS expression levels are important for lipid homeostasis [96]. Xu et al. also showed that insulin inhibits fatty acid oxidation by downregulating the activity of enzymes like carnitine palmitoyltransferase 1, reducing the transport of fatty acids into mitochondria for β-oxidation [96]. This process promotes lipid storage over lipid breakdown. Studies using long-lived mutant nematodes (*Caenorhabditis elegans*) have also demonstrated the relationship between lifespan and lipid metabolism. Long-lived mutant genes *age-1* and *daf-2* in these nematodes are closely related to the insulin signaling pathway and have been useful in aging studies [97,98]. Since insulin acts as a major anabolic agent that promotes the synthesis and storage of glucose, proteins, and lipids, it is imperative to research the interplay between DR, lipid metabolism, and the aging process with the regulation of insulin signaling.

### 2.4. Cell Membrane Lipid and Health

Lipid metabolism is very important in that it not only functions as a mechanism of metabolic energy storage but also has a close impact on cell membrane function. Lipid metabolism primarily involves the formation of membranes from non-lipid sources. Moreover, it was observed that a restricted diet increased the fluidity of erythrocyte membranes and had a protective effect on cell membranes in rats [99,100]. Considering the obvious evidence that aged individuals have reduced cell membrane fluidity [101], these results indicate that DR has beneficial effects on cell membranes. Also, the maximum lifespan of the mammals and the membrane lipid peroxidation index were inversely correlated [102]. Lipid peroxidation increases oxidative stress in cell membranes, causes cell damage, and can finally lead to cell death. At the organism level, the accumulation of oxidative stress promotes aging by inducing diseases such as cancer and cardiovascular disease and the production of mitochondrial reactive oxygen species (ROS). Hulbert used the phrase “membrane pacemaker theory” to emphasize that lipid peroxidation and its reactants determine the lifespan of organisms [103]. Hulbert showed this correlation not only in mammals having shortened lifespans due to feeding on toxic substances but also in mammals having an extended lifespan through DR. A study that identified genetic targets in long-lived mammals using a phylogenetic approach found that mammals with genes that bind to lipids selectively live longer [104]. Studies in birds have shown that parrots that live long produce more monounsaturated fatty acid (MUFA), less polyunsaturated fatty acid (PUFA), and lower cardiac phospholipid peroxidation index compared to short-lived birds [105]. Additionally, studies in birds and mice with the same body size and mass have shown that birds with larger, slower-beating hearts and membrane compositions are less susceptible to damage due to lipid peroxidation and live longer [106]. In the case of nematodes, it has been shown that the fatty acid composition in lipids is an important determinant of longevity in nematodes rather than the total lipid contents [107]. Knocking down the fat-4 gene (encoding the ∆5 desaturase enzyme that enables the conversion of 20:3*n*-6 → 20:4*n*-6 and 20:4*n*-3 → 20:5*n*-3) resulted not only in remarkable resistance to hydrogen peroxide exposure but also in significant lifespan extension, confirming that fat composition is involved in extending the lifespan [108]. A recent study using *C. elegans* fed *Escherichia coli* (*E. coli*) mutants depleted of intracellular glucose proved that glucose restriction increases the lifespan of worms by promoting membrane fluidity in the peripheral tissues [109]. Taken together, lipid metabolism plays an important role in both energy storage and cell membrane function, suggesting that DR may inhibit aging by regulating lipid metabolism and preventing cell damage in a variety of organisms.

### 2.5. Commensal Bacteria and Lipid Profile

Recent studies have shown evidence that interactions between host and gut bacteria affect the aging of the host [110,111,112,113]. Most commensal bacteria live in the intestines of the host, and the commensal gut microbiome changes dynamically depending on the host’s diet type [114]. DR was found to have a beneficial effect on the host not only through changes in the individual’s molecular mechanisms as mentioned above, but also through changes in the intestinal microbial flora [115,116,117]. For instance, in a study on obese and overweight adult subjects, DR, especially intermittent calorie restrictions, maintained stable gut microbiota involving *Akkermansiaceae*, *Christensenellaceae*, and *Tanerellaceae* and improved subjects’ health status by inducing weight loss [116]. A study conducted among overweight and obese Chinese women demonstrated that both low-carbohydrate (LC) and CR diets effectively reduced weight and fat mass. However, only the LC diet significantly improved dyslipidemia. These improvements were associated with changes in gut microbiota, as the two diets altered microbial structure and co-abundance gene clusters to varying degrees. Notably, the *Bacteroidetes*/*Firmicutes* ratio, a marker of gut microbial health, increased significantly in the LC diet group but not in the CR group, highlighting the distinct impact of dietary composition on gut microbiota. These microbiota changes contributed to the improved lipid profiles observed in the participants [118]. Furthermore, the gut microbiota is different between mice fed diets that are high or low in fat [119,120,121]. Just as the host’s diet affects the gut microbiota, many studies have shown that the commensal microbiota affects the lipid metabolism of the host by transforming, synthesizing, and breaking down the lipids. In mice, the levels of cholesterol, fatty acids, triglycerides, and phosphatidylcholine in the serum were reduced in a conventionally raised condition (with microbiota) compared to a germ-free condition (without microbiota), and these levels were affected by the presence of commensal bacteria [122]. Notably, the commensal bacterial flora plays pivotal roles in the regulation of host cholesterol and sphingolipid homeostasis [123]. Also, metabolites produced by commensal bacteria such as short-chain fatty acids (SCFA), secondary bile acids, and lipopolysaccharides affect host lipid metabolism [124]. Dietary methionine restriction, a form of dietary restriction, has been shown to extend lifespan, improve lipid metabolism, suppress inflammation, change the gut microbiota, and enhance gut immunity in a variety of animals [125,126,127]. A recent study revealed the interconnected role of dietary methionine restriction in regulating lipid metabolism, inflammatory responses, and gut microbiota, demonstrating that it reduces fat accumulation and systemic inflammation through gut microbial alterations in middle-aged mice fed high-fat diets [128]. Dysbiosis of commensal microbiota can induce disturbed homeostasis of the lipid metabolism, which leads to an increase in the risk of gut inflammation, which is a critical factor in the development of various age-related conditions and diseases [129]. Moreover, the modulation of gut microbiota by probiotics treatment resulted in lipid profile changes and enhanced insulin sensitivity in mice with Alzheimer’s disease (AD), a representative age-related neurodegenerative disease. This result indicates that the symbiosis induced by probiotics positively changed the lipid composition in mice with AD [130]. Disruption of cerebral cholesterol metabolism is a hallmark of both aging and AD, with lipid-lowering agents being associated with a reduced risk of neurodegenerative disorders. Recent studies have shown that oral supplementation with multi-strain probiotics reduced amyloid beta aggregation and brain damage in AD transgenic mouse models, leading to improved cognitive function [131]. Recently, there has been active research on the host aging-commensal bacteria interaction. Despite the growing evidence linking gut microbiota, lipid metabolism, and aging, several knowledge gaps remain. Great progress is expected in the future on the elucidation of the relationship between host aging, commensal bacteria, and lipid metabolism. Future research should focus on elucidating the specific roles of individual microbial species and their metabolites in lipid regulation and age-related metabolic diseases. Long-term clinical studies are needed to understand the sustained effects of dietary modulation and probiotic interventions on gut microbiota and lipid homeostasis. Furthermore, exploring the interactions between diet, gut microbiota, and systemic inflammation may offer novel strategies for managing metabolic diseases such as obesity, diabetes, and cardiovascular disease.

### 2.6. Intermittent Fasting and Lipid Profile

Building on the insights from DR, intermittent fasting (IF) similarly exerts beneficial effects on lipid metabolism by modulating key pathways. IF is a dietary manipulation that involves cycling between periods of feeding and fasting [132]. There are different approaches to IF, such as alternate-day fasting (fasting every other day or 5 days of feeding followed by 2 days fasting) and time-restricted eating (16 h of fasting followed by 8 h feeding window). IF is distinct in that it focuses on the timing of food intake rather than the number of calories consumed. Both DR and IF alter the activities of common key metabolic pathways, such as sirtuins, TOR, and IIS pathways [133,134]. As discussed in the section on sirtuins, DR-induced SIRT1 activation plays a critical role in promoting β-oxidation and enhancing fat utilization. Similarly, IF has been shown to elevate *Sirt1* mRNA expression and activity in mouse liver and neuronal retina progenitor cells [135], driving lipid catabolism during fasting periods. Additionally, IF mirrors DR by inhibiting mTORC1, facilitating a shift from anabolic to catabolic processes and inducing autophagy. For instance, in a study using rats with experimentally induced NAFLD, IF significantly reduced liver injury markers, including serum triglycerides (TG), alanine transaminase (ALT), and aspartate transaminase (AST), compared to control groups [136]. Ma et al. demonstrated that IF could mitigate cardiomyopathy by normalizing desmin localization through autophagy-dependent mechanisms via reduced mTOR activation. Nevertheless, the inhibitory effects of IF on mTOR signaling in WAT have been little explored [137,138]. In the IIS pathway, IF promotes triacylglycerol deposition in white adipose tissue by regulating insulin and lipid-associated gene expression, such as *Fsp27*/*Cidec* [139]. Additionally, IF improves glycemia, lipid metabolism, and insulin resistance in mice subjected to dietary overload with high-fat or high-fructose diets [140]. Conversely, IF demonstrates dual effects by reducing fat mass while impairing hepatic insulin signaling and lipid metabolism in young rats fed high-protein and high-fat diets [141]. These findings underscore the complexity of IF’s impact on IIS and lipid homeostasis. These shared molecular pathways highlight the convergence of DR and IF in their metabolic benefits, underscoring their potential to improve lipid metabolism and extend the healthspan. IF leads metabolic switching, stress resistance, and improved glucose metabolism and achieves many benefits of DR without sustained calorie reduction. Therefore, lipid changes are observed even through IF without calorie changes. IF promotes lipid oxidation, particularly during fasting periods, as it enhances the breakdown of fat stores, contributing to fat loss and reducing visceral fat accumulation [142]. IF may preferentially reduce ectopic fat, such as fat stored in the liver, which is beneficial for preventing conditions like fatty liver disease and improving overall metabolic health [143]. Also, IF reduce levels of total cholesterol, low-density lipoprotein, and triglycerides while increasing high-density lipoprotein levels. This improvement is observed in both healthy individuals, obesity, and dyslipidemia patients [144,145,146]. A recent study explored how IF affects lipid metabolism in obese mice. Mice on an alternate day fasting regimen showed weight loss, lower serum lipid levels, and reduced liver fat accumulation compared to those with continuous feeding. IF also decreased metabolic endotoxemia and altered gut microbiota, increasing diversity and shifting the balance toward beneficial bacteria (e.g., reduced the *Firmicutes*/*Bacteroidetes* ratio and increased the relative abundance of *Allobaculum* in the intestinal flora). Also, CR or IF reduces neuroinflammation in the hypothalamus and hippocampus of rodents exposed to bacterial lipopolysaccharide [147,148]. Overall, IF improved lipid metabolism, reduced fat storage, and supported healthier gut flora [149]. The clinical study involving 470 NAFLD patients with IF showed reductions in body weight, fat mass, and liver fat (hepatic steatosis) and alanine aminotransferase levels, all markers of liver damage. However, fat-free mass and HDL cholesterol levels showed no significant changes [150]. These findings suggest that the metabolic benefits of IF closely mirror those of DR, reinforcing the importance of shared molecular pathways in extending the healthspan. Given that the molecular mechanisms of IF closely resemble those of DR, it is hypothesized that the effects of IF on lipid profiles will be largely similar to those observed in DR. However, research specifically addressing the long-term effects of IF remains limited. Future studies should actively explore the molecular-level impacts of IF on lipid profiles, with an emphasis on elucidating its long-term outcomes.

## 3. Conclusions

**Figure 2:**
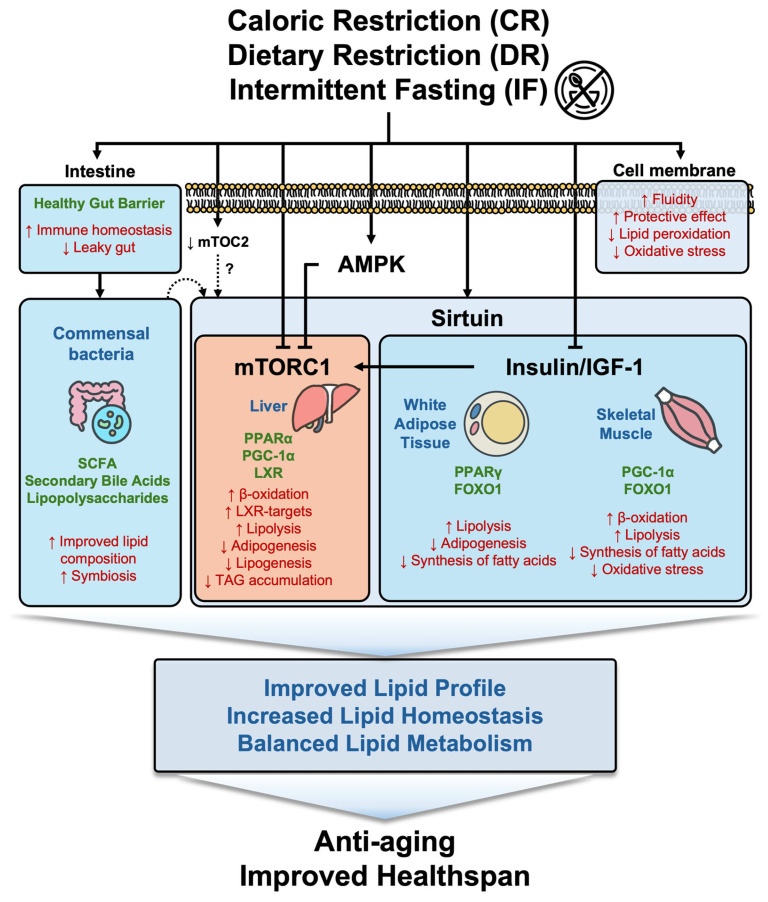
Effects of dietary restriction on lipid profile changes and healthspan. This figure summarizes the molecular mechanisms by which DR improves lipid profile and enhances the healthspan. DR impacts multiple pathways, including sirtuins, mTORC1, and the insulin/IGF-1 signaling pathway, which regulate lipid metabolism in the liver, adipose tissue, and skeletal muscle. DR also promotes a healthy gut barrier and modulates commensal microbiota, enhancing short-chain fatty acid (SCFA) production and immune homeostasis. Through increased β-oxidation, reduced lipogenesis, and enhanced oxidative stress resistance, DR maintains lipid homeostasis, reduces oxidative damage, and improves membrane fluidity. These coordinated effects result in balanced lipid metabolism, improved health outcomes, and anti-aging benefits.

In conclusion, the studies explored in this review describe the intricate and significant role of lipid metabolism in DR in relation to aging (Figure 2). DR, as a potent intervention for slowing aging, has shown promising effects in modulating lipid metabolism, thereby influencing aging across various species. Our review highlights that, despite significant progress in understanding the interplay between DR and lipid metabolism, several critical knowledge gaps persist, underscoring the need for further research. In conclusion, sirtuins play a pivotal role in lipid metabolism by promoting lipid oxidation, reducing fat storage, and enhancing energy homeostasis across various tissues through NAD^+^-dependent deacetylation. Their activation, along with the regulation of autophagic pathways, positions sirtuins as promising therapeutic targets for metabolic diseases such as obesity and NAFLD. While the sirtuin family is central to lipid homeostasis, their precise roles, particularly in connection to autophagy, remain incompletely understood. Expanding autophagy-related studies is expected to provide valuable insights into how cellular degradation processes intersect with lipid regulation and aging. Additionally, the mTOR and insulin signaling pathways, which promote lipogenesis under anabolic conditions, can be modulated through IF or DR to shift metabolism toward fat utilization. Given the distinct contributions of mTORC2 to cellular signaling and metabolic processes, future research should aim to differentiate and define the specific actions of mTORC1 and mTORC2 in lipid regulation, providing a more comprehensive understanding of these pathways. The role of insulin signaling, a key anabolic regulator, also demands further exploration in the context of DR, lipid metabolism, and aging. Understanding how DR influences insulin-mediated lipid synthesis and storage processes, as well as its broader impact on aging, will be crucial for developing targeted interventions. IF, in particular, mirrors the benefits of DR by improving lipid profiles and reducing visceral fat without sustained calorie reduction. However, while IF has shown promise in modulating lipid profiles and improving metabolic health, studies addressing its long-term effects remain limited. Future research should focus on elucidating the molecular mechanisms underlying IF’s benefits and assessing its sustained impact on lipid metabolism and aging-related processes. Moreover, the interaction between gut microbiota and host lipid metabolism further highlights the potential for future research into diet-microbiota modulation to improve metabolic health and combat age-related diseases. However, there are significant gaps in understanding the specific roles of microbial species and their metabolites in lipid regulation. Long-term clinical studies investigating the sustained effects of dietary modulation and probiotic interventions on gut microbiota and lipid homeostasis are needed. The gut–brain axis has emerged as a promising area of research with potential applications for treating neurological diseases. These findings suggest that modulating the gut microbiota through DR could positively influence lipid metabolism, thereby enhancing brain health. However, the specific role of lipid metabolism in the gut–brain axis under DR remains unexplored, highlighting the need for further studies to bridge this knowledge gap. By addressing these gaps, future studies can provide a more holistic understanding of how DR and related interventions influence lipid metabolism and aging. This will not only advance fundamental scientific knowledge but also pave the way for novel therapeutic strategies targeting age-related metabolic diseases. Overall, the interplay between diet, lipid metabolism, and aging represents a frontier with untapped potential, with the possibility of promising advances in understanding and managing the aging process.
